# Relationship of Baseline Hemoglobin Level with Serum Ferritin, Postphlebotomy Hemoglobin Changes, and Phlebotomy Requirements among *HFE* C282Y Homozygotes

**DOI:** 10.1155/2015/241784

**Published:** 2015-08-26

**Authors:** Seyed Ali Mousavi, Faiza Mahmood, Astrid Aandahl, Teresa Risopatron Knutsen, Abid Hussain Llohn

**Affiliations:** Department of Immunology and Transfusion Medicine, Akershus University Hospital, University of Oslo, Norway

## Abstract

*Objectives*. We aimed to examine whether baseline hemoglobin levels in C282Y-homozygous patients are related to the degree of serum ferritin (SF) elevation and whether patients with different baseline hemoglobin have different phlebotomy requirements. *Methods*. A total of 196 patients (124 males and 72 females) who had undergone therapeutic phlebotomy and had SF and both pre- and posttreatment hemoglobin values were included in the study. *Results*. Bivariate correlation analysis suggested that baseline SF explains approximately 6 to 7% of the variation in baseline hemoglobin. The results also showed that males who had higher (≥150 g/L) baseline hemoglobin levels had a significantly greater reduction in their posttreatment hemoglobin despite requiring fewer phlebotomies to achieve iron depletion than those who had lower (<150 g/L) baseline hemoglobin, regardless of whether baseline SF was below or above 1000 *µ*g/L. There were no significant differences between hemoglobin subgroups regarding baseline and treatment characteristics, except for transferrin saturation between male subgroups with SF above 1000 *µ*g/L. Similar differences were observed when females with higher (≥138 g/L) baseline hemoglobin were compared with those with lower (<138 g/L) baseline hemoglobin. *Conclusion*. Dividing C282Y-homozygous patients into just two subgroups according to the degree of baseline SF elevation may obscure important subgroup variations.

## 1. Introduction

Hemochromatosis due to mutations in the hemochromatosis Fe (*HFE*) gene is the most common, autosomal recessive, form of inherited iron-overload disorder among people originating from northwestern Europe. This form of hereditary hemochromatosis (HH) is due to excessive intestinal iron absorption. As there is no regulated pathway for iron excretion in humans this leads to accumulation of iron in parenchymal cells of different tissues, particularly the liver, pancreas, and heart [1, 2].* HFE*-associated HH is characterized biochemically by elevated transferrin saturation (TS), a measure of iron availability for erythropoiesis, and high levels of serum ferritin (SF), an iron storage protein reflecting body iron stores [3]. Two missense mutations, C282Y (substitution of tyrosine for cysteine at position 282) and H63D (substitution of aspartic acid for histidine at position 63), are the most frequent (~8% and ~15%, resp.) among individuals of northern European ancestry [4]. However, the majority of* HFE*-associated HH patients (>90%) are homozygous for the C282Y variant, about 6% are C282Y/H63D compound heterozygotes, and less than 1% of reported cases are H63D homozygotes [1, 5, 6].

Therapeutic phlebotomy, when this is feasible, has been the preferred treatment for clinical management of this patient population. Depletion of the excess iron by phlebotomy consists of removing one unit of blood (approximately 450 mL + 30 mL for laboratory measurements) until SF levels are <50 *μ*g/L and, when initiated early, will prevent severe iron overload and iron-related disease such as liver cirrhosis and cardiovascular problems [7].

The present study was prompted by our observation that some C282Y-homozygous patients with higher baseline hemoglobin had lower posttreatment hemoglobin levels in spite of requiring fewer phlebotomies than patients with lower baseline hemoglobin. However, this informal observation was not directly investigated at our institution. Moreover, to our knowledge, whether baseline hemoglobin level varies depending on the degree of SF elevation has not been properly addressed in the literature about C282Y-homozygous patients. In this study, we first examined whether there is a relationship between baseline hemoglobin and the degree of SF elevation among C282Y-homozygous patients. We then evaluated the effect of therapeutic phlebotomy on changes in posttreatment hemoglobin levels among subgroups of C282Y homozygotes.

## 2. Methods

### 2.1. Study Subjects

This was a retrospective study conducted at Akershus University Hospital blood bank in Norway. In addition to collecting blood for transfusion, the blood bank provides services for individuals that require therapeutic phlebotomy. After obtaining approval from the Institutional Review Board, patient databases were used to collect relevant data of HH patients who had undergone therapeutic phlebotomy during 1996 to 2013. For this analysis, patients were included if they (1) had known gender, (2) had available data on age at baseline, (3) had undergone testing for* HFE* mutations and were confirmed to be either homozygote (C282Y/C282Y) or compound heterozygote (C282Y/H63D), (4) had elevated baseline SF (>200 *μ*g/L in females and >300 *μ*g/L in males), and (5) had recorded venous hemoglobin values at baseline and at the last phlebotomy when their SF was below 50 *μ*g/L (referred to as posttreatment hemoglobin).

Genotype data were available on 254 patients. Of these, 32 (12.6%) had incomplete baseline data and were therefore excluded. Five C282Y-homozygous males were excluded from analysis as a result of (1) alcohol abuse (*n* = 2), (2) cirrhosis (*n* = 1), and (3) blood donation (*n* = 2 prior blood donors), because these conditions are capable of affecting SF levels. Three H63D-homozygous males were also excluded due to uncertainty about whether they were true HH subjects, leaving 214 patients for this analysis. Patients with low SF (females, between 200 and 1000 *μ*g/L; males, between 300 and 1000 *μ*g/L) and high (above 1000 *μ*g/L) SF levels were included in order to increase the statistical power for demonstrating the presence (or lack) of an association. Transferrin-iron saturation could not be calculated for all of these patients because of missing transferrin or serum iron data for 14% (30/214) of them. No patients were excluded from the analysis due to missing TS values, because this would yield small sample sizes. Transferrin saturation was calculated as 4 × (serum iron [in *μ*mol/L])/(serum transferrin [in g/L]) or by dividing the serum iron by the total iron-binding capacity and multiplying by 100.

### 2.2. Statistical Analysis

Unpaired *t*-test was used to compare means between continuous variables and Pearson's chi-square or Fisher's exact test to compare differences among group proportions. Since serum ferritin values and the number of phlebotomies were not normally distributed, mean values were compared after logarithmic transformation. Paired *t*-test and Wilcoxon signed-rank test were used comparing baseline and posttreatment hemoglobin levels. Pearson's correlation analysis was performed to assess the relationship between baseline levels of hemoglobin and other baseline characteristics, and those significantly associated with baseline hemoglobin were included in multiple linear stepwise regression analysis. Missing TS data were imputed by the mean of the two straddling values. In all cases, a *p* value of <0.05 was considered statistically significant. Because of gender-specific differences in baseline characteristics, separate analyses were performed for male and female C282Y homozygotes. Statistical analyses were performed using SPSS software version 21 (Chicago, IL, USA).

## 3. Results

### 3.1. Baseline Characteristics of Patients

The final study population consisted of 214 patients; of these 142 (66.3%) were males and 72 (33.7%) were females. Baseline characteristics of patients in each genotype group according to gender are summarized in Table 1. The majority of patients (*n* = 196, 91.6%) were homozygous for C282Y, as expected. Of these, 124 (63.3%) were males and 72 (36.7%) were females. Among C282Y-homozygous patients, males on average were 7.3 years younger than females (*p* < 0.0001) but they had significantly higher mean SF (*p* < 0.0001) and slightly higher mean TS levels (*p* = 0.17) than females. Compound-heterozygous (C282Y/H63D) males had significantly lower SF (*p* = 0.012) and TS (*p* < 0.0001) levels than C282Y-homozygous males.

### 3.2. Differences in Mean Baseline Hemoglobin Levels across Genotype Groups

Most patients had baseline hemoglobin levels that fell within the reference range for hemoglobin (Table 1). Among C282Y-homozygous patients, males had significantly higher mean hemoglobin values than females (*p* < 0.001), as might be expected because of a physiologically higher hemoglobin concentration in men. Compound-heterozygous males had a mean hemoglobin value that was slightly higher than that of C282Y-homozygous males but the difference was not statistically significant.

### 3.3. Relationship between Baseline Hemoglobin and SF Levels

Further analysis of the data, which was limited to C282Y-homozygous patients, revealed that patients with lower SF had higher baseline hemoglobin. These patients were therefore categorized as having low (<1000 *μ*g/L; mean 658 ± 170 *μ*g/L; range 316–972 *μ*g/L) or high (>1000 *μ*g/L; mean 1680 ± 856 *μ*g/L; range 1030–4000 *μ*g/L) SF in order to compare their baseline hemoglobin levels. Males with SF levels <1000 *μ*g/L (68.5%, 85/124) had higher mean hemoglobin concentration than those with SF levels >1000 *μ*g/L (151.9 ± 8.4 versus 147.1 ± 12.6 *μ*g/L; *p* = 0.014). Similarly, females with SF <1000 *μ*g/L (84.7%, 61/72) had higher mean hemoglobin concentration than those with SF levels >1000 *μ*g/L (139.0 ± 8.1 versus 134.0 ± 6.6 *μ*g/L), although the difference was of borderline significance (*p* = 0.059) (see Table 3). The unadjusted odds ratio (OR) of having SF >1000 *μ*g/L for males versus females was 2.54 (95% confidence interval [CI]: 1.2–5.4; *p* = 0.012).

Correlation analysis (Table 2) showed that baseline hemoglobin in C282Y-homozygous males was negatively correlated with age (*r* = −0.29, *p* = 0.001), log-SF (*r* = −0.25, *p* = 0.006), and TS (*r* = −0.23, *p* = 0.01), indicating that increased values of these variables are associated with lower levels of baseline hemoglobin. When significant determinants of baseline hemoglobin (as dependent variable) were examined simultaneously in a multiple linear regression model, age (*β*, −0.29, *p* = 0.001) and TS (*β*, −0.23, *p* = 0.007) remained significant but log-SF did not retain statistical significance. The overall *R* ^2^ for the model was 0.123 (*p* = 0.001). However, in a regression model that also included the age by TS interaction, age and TS did not remain independently significant (*β*, 0.39, *p* < 0.0001 for the interaction term). The overall *R* ^2^ for the model was 0.144 (*p* < 0.0001), indicating that approximately 14.4% of the variability in baseline hemoglobin values was accounted for by the effect of combined variation of age and TS. In females, only log-SF was correlated with baseline hemoglobin (*r* = −0.27, *p* < 0.01), indicating that baseline SF may explain approximately 7.3% of the variability in baseline hemoglobin values.

### 3.4. Effect of Therapeutic Phlebotomy on Posttreatment Hemoglobin Levels

Table 3 shows mean (95% CI) decreases and percentage changes in posttreatment hemoglobin from baseline among male and female patients. Among C282Y-homozygous males, the mean hemoglobin decreased by 6.0 g/L (*p* < 0.0001, paired *t*-test). Compared with baseline values, posttreatment hemoglobin levels decreased in 93 patients (75%), increased in 30 patients (23.4%), and remained the same in one patient. Similar results were also observed among C282Y-homozygous females; the mean hemoglobin decreased by 5.1 g/L (*p* < 0.0001, paired *t*-test); posttreatment hemoglobin levels decreased in 52 patients (72.2%), increased in 17 patients (23.6%), and remained unchanged in 3 patients. Among compound-heterozygous males, the mean hemoglobin decreased by 9.9 g/L (*p* < 0.0001, paired *t*-test); one patient (4.8%) had a rise in posttreatment hemoglobin and 2 patients had no change in their hemoglobin levels.

To further examine the relationship between baseline hemoglobin, serum ferritin, and changes in posttreatment hemoglobin levels, C282Y-homozygous patients in each of the two SF categories were divided into those with hemoglobin <150 and ≥150 g/L or <138 and ≥138 g/L based on mean baseline hemoglobin values for male and female patients, respectively. The results of this subanalysis are shown in Table 3.

Among the subgroups with higher (≥150 g/L) hemoglobin, the percentage change in hemoglobin from baseline for males with low (<1000 *μ*g/L) SF was −5.3% and for those with high (>1000 *μ*g/L) SF it was −5.7% compared, respectively, with −2.6% and −1.3% in the two subgroups with lower (<150 g/L) hemoglobin (*p* = 0.038 and *p* = 0.014, resp.). Among the subgroups with high (>1000 *μ*g/L) SF, males with higher (≥150 g/L) hemoglobin had lower TS than those with lower (<150 g/L) hemoglobin (80.9 ± 12% versus 91.9 ± 6.6%, *p* = 0.002), but no other differences between SF subgroups were significant (Table 4).

A similar pattern of results was observed among females: the percentage change for females with higher (≥138) hemoglobin and low (<1000 *μ*g/L) SF was −5.5%, significantly different from −2.1% among those with lower (<138 g/L) hemoglobin and low (<1000 *μ*g/L) SF (*p* = 0.020). The percentage change for females with higher (≥138 g/L) hemoglobin and high (>1000 *μ*g/L) SF was −3.7%, but there was basically no difference between baseline and posttreatment hemoglobin for females with lower (<138 g/L) hemoglobin and high (>1000 *μ*g/L) SF, except for the wider standard deviation in their posttreatment hemoglobin values (Table 3). There were no statistically significant differences in baseline characteristics between the two subgroups within each SF category (Table 4).

There was a higher proportion of males with baseline hemoglobin ≥150 g/L among patients with SF <1000 *μ*g/L than among patients with SF >1000 *μ*g/L (61% versus 41%, OR 2.2, 95% CI, 0.99–4.7; *p* = 0.049); the corresponding proportions for females with baseline hemoglobin ≥138 g/L within the low and high SF categories were 57% and 27%, respectively (OR 3.6, 95% CI, 0.9–14.8, *p* = 0.10 by Fisher's exact test).

The results from this subanalysis indicate that C282Y-homozygous patients with higher baseline hemoglobin had a larger decrease in their posttreatment hemoglobin levels than those with lower baseline hemoglobin levels regardless of whether baseline SF was above or below 1000 *μ*g/L. The significant higher decrease in posttreatment hemoglobin levels among patients with higher baseline hemoglobin relative to those with lower hemoglobin levels was not because they had higher phlebotomy requirements. Among males within both SF categories, the mean number of phlebotomies required to achieve iron depletion among the subgroups with higher (≥150 g/L) baseline hemoglobin was lower (14.3 ± 5.2 [range 6–35 phlebotomies] and 26.7 ± 11.3 [range 7–52 phlebotomies], resp., for low and high SF categories) than the subgroups with lower (<150 g/L) baseline hemoglobin (18.8 ± 8.1 [range 8–35 phlebotomies] and 39.1 ± 22.2 [range 14–94 phlebotomies]; *p* = 0.038 and *p* = 0.06, resp., for low and high SF categories). Among females within the low SF category, the mean number of phlebotomies required to achieve iron depletion was similar between the two hemoglobin subgroups (*p* = 0.64), whereas, among those within the high SF category, the mean number of phlebotomies required to achieve iron depletion among the subgroup with higher (≥138 g/L) baseline hemoglobin was significantly lower than the mean among the subgroup with lower (<138 g/L) baseline hemoglobin (20.7 ± 10.3 [range 12–32 phlebotomies] versus 37.3 ± 13.9 [range 23–68 phlebotomies]; *p* = 0.036).

To ensure that subgroup differences in posttreatment hemoglobin changes were not due to differences in treatment intensity at the end of therapeutic phlebotomy and to control for the degree of phlebotomy-induced iron depletion, we compared the average time interval between the penultimate phlebotomy and the last phlebotomy and the mean posttreatment SF value for each subgroup.

The mean time intervals between penultimate phlebotomy and the last phlebotomy are shown in Table 4. Among males, the mean time intervals for subgroups with higher (≥150 g/L) baseline haemoglobin were somewhat, though not significantly, shorter (15.7 ± 6.12 [range 7–34 days] and 13.6 ± 4.08 [range 7–21 days], resp., for low and high SF categories) than those of subgroups with lower (<150 g/L) baseline haemoglobin (16.1 ± 4.10 [range 7–28 days] and 14.4 ± 3.41 [range 7–21 days], resp., for low and high SF categories). The mean time interval for the female subgroup with SF <1000 *μ*g/L and with higher (≥138 g/L) baseline haemoglobin was slightly shorter than that of the female subgroup with SF <1000 *μ*g/L and with lower (<138 g/L) baseline haemoglobin (15.8 ± 5.19 [range 7–28 days] versus 17.0 ± 5.03 [range 7–28 days]). The female subgroup with SF >1000 *μ*g/L and with higher (≥138 g/L) baseline haemoglobin had a slightly longer mean interval than that of the female subgroup with SF >1000 *μ*g/L and with lower (<138 g/L) baseline haemoglobin (18.7 ± 4.04 [range 14–21 days] versus 16.1 ± 4.26 [range 10–21 days]). These differences were not significant (Table 4).

There were no statistically significant differences in the levels of posttreatment SF between the two hemoglobin subgroups within each SF category (Table 4). No patients in either hemoglobin subgroup within each SF category had developed iron deficiency (SF <20 *μ*g/L) at the last phlebotomy. Among males with SF <1000 *μ*g/L, posttreatment SF levels ranged from 27 to 50 *μ*g/L in the subgroup with higher (≥150 g/L) baseline haemoglobin and from 32 to 49 *μ*g/L in the subgroup with lower (<150 g/L) baseline haemoglobin, whereas they ranged from 35 to 48 *μ*g/L in both subgroups with SF >1000 *μ*g/L. Among females with SF <1000 *μ*g/L, posttreatment SF levels ranged from 29 to 52 *μ*g/L in the subgroup with higher (≥138 g/L) baseline haemoglobin and from 31 to 49 *μ*g/L in the subgroup with lower (<138 g/L) baseline haemoglobin, whereas, among those with SF >1000 *μ*g/L, they ranged from 41 to 45 *μ*g/L in the subgroup with higher (≥138 g/L) baseline haemoglobin and from 38 to 48 in the subgroup with lower (<138 g/L) baseline haemoglobin.

The time interval between the penultimate phlebotomy and the last phlebotomy for compound-heterozygous males combined was an average of 16.2 ± 7.6 days (range 7–28 days) and their posttreatment SF levels averaged 42.4 ± 5.8 *μ*g/L (range 25–48 *μ*g/L). A comparison between these males and C282Y-homozygous males with comparable SF levels (see Tables 1 and 4) indicates that compound-heterozygous males had larger decrease in posttreatment hemoglobin levels in spite of requiring, on average, fewer phlebotomies than C282Y homozygotes with similar baseline SF levels (Table 3).

### 3.5. Determinants of Phlebotomy Requirements

Bivariate correlation of log number of phlebotomies with baseline variables in C282Y-homozygous patients is shown in Table 2. To examine whether baseline hemoglobin independently predicts the number of phlebotomies required to induce iron depletion, multiple linear regression analysis was performed, adjusting for age (only in males), log-SF, and TS. In males, the multiple regression analysis showed that Log-SF (*β*, 0.62, *p* < 0.0001), baseline hemoglobin (*β*, −0.25, *p* < 0.0001), and age (*β*, 0.17, *p* = 0.004) were significantly associated with the log number of phlebotomies. Baseline TS was not a significant predictor, however. The overall *R* ^2^ for the model was 0.636 (*p* < 0.0001). The results indicated that an increase in SF of 42 *μ*g/L was associated with one additional unit of blood to be removed to achieve iron depletion, whereas a decrease in baseline hemoglobin of 0.45 g/L was associated with a decrease of phlebotomy requirements of one unit of blood. The regression model predicting the number of phlebotomies in females showed that log-SF was significantly and positively (*β*, 0.62, *p* < 0.0001) associated with the log number of phlebotomies but baseline hemoglobin and TS did not remain. The overall *R* ^2^ for the model was 0.528 (*p* < 0.0001). In females, an increase in SF of 33 *μ*g/L was associated with one additional unit of blood to be removed to achieve iron depletion.

## 4. Discussion

In this retrospective study among a cohort of C282Y-homozygous patients, we examined the relation between baseline SF and hemoglobin. In addition, we evaluated the effect of therapeutic phlebotomy on changes in posttreatment hemoglobin levels among subgroups with higher and lower baseline hemoglobin. Our results showed that, in C282Y-homozygous patients, lower baseline serum ferritin was associated with higher baseline hemoglobin levels. In bivariate analysis of C282Y-homozygous males, there was weak but significant inverse relationship between baseline hemoglobin and log-SF, age, and TS. However, the association between hemoglobin and log-SF did not remain in a multiple regression model adjusting for age and TS. The adjusted model described that more than 85% of the variability in baseline hemoglobin was accounted for by variables not included in the model. In C282Y-homozygous females, baseline hemoglobin correlated negatively only with log-SF, explaining about 7.3% of the variability in baseline hemoglobin. Taken together, these results suggest that differences in serum iron markers' baseline levels are inadequate to explain much of the variability in baseline hemoglobin. Or, in other words, we do not understand a great deal about the determinants of baseline hemoglobin in this patient population, perhaps because the level of hemoglobin production in these patients is strongly influenced by other factors such as genetic modifiers (see below).

Further subanalysis showed that C282Y-homozygous males who had higher baseline hemoglobin (≥150 g/L) had significantly higher decrease in posttreatment hemoglobin levels compared to those with lower baseline hemoglobin (<150 g/L), regardless of baseline SF category. Similar differences were observed when comparing high (≥138 g/L) and low (<138 g/L) hemoglobin subgroups among C282Y-homozygous females. Comparisons of baseline variables and treatment characteristics between hemoglobin subgroups within each SF category did not indicate any significant group differences except for the mean values of baseline TS within the group with high SF (>1000 *μ*g/L).

Several studies in subjects with* HFE* gene mutations have found significant increases in red blood cell parameters including hemoglobin and mean corpuscular volume as compared with normal controls [8–11]. The elevated hemoglobin observed in C282Y-homozygous subjects has been ascribed to increased uptake of transferrin-bound iron by erythroid precursors, possibly through upregulation of transferrin receptor 1 (TfR1) [8]. Assuming mean hemoglobin concentrations in normal men and women are 150 g/L and 140 g/L, respectively, that is, 20 g/L higher than the cutoff level for anemia [12], our results show that compound-heterozygous males as a whole had a mean hemoglobin level that was ~3.2 g/L above the normal mean value. C282Y-homozygous males as a whole had a mean hemoglobin level that was only slightly higher than the average, whereas C282Y-homozygous females as a whole had a mean hemoglobin level that was lower than the average. However, about 54% (67/124) of C282Y-homozygous males in our patient population had hemoglobin levels that were 7.5 to 10.3 g/L above the normal mean value for men compared with about 43% (30/72) females who had hemoglobin levels that were 2 to 4.3 g/L above the normal mean value for women.

The retrospective nature of the study does not allow us to explain the cause(s) of differences in baseline hemoglobin levels at a given SF among C282Y-homozygous patients or to definitively determine why those with higher baseline hemoglobin have greater decrease in posttreatment hemoglobin despite requiring lower phlebotomy requirements to achieve iron depletion. However, we can speculate on some potential explanations for these observations. Iron overload associated with* HFE* mutations has been shown to result from inadequate production (relative to the degree of iron overload) of the hepatic peptide hormone hepcidin [13, 14]. Circulating hepcidin is the central regulator of systemic iron metabolism by directly binding and downregulating the cell surface iron transporter ferroportin-1 (FPN1) and reducing/inhibiting iron efflux from duodenal enterocytes, reticuloendothelial macrophages, and hepatocytes [15]. In hepatocytes, the HFE protein appears to be part of an iron-sensing complex which has a key role in regulating hepcidin expression. This complex also involves TfR2, bone morphogenetic protein 6 (BMP-6), the BMP receptor complex composed of activin-like kinase receptor 2 (ALK2) and ALK3 and its intracellular signaling pathway (Smad proteins), and hemojuvelin, which is a coreceptor for BMP-6 signaling [16–21].

The normal* HFE* gene encodes a transmembrane glycoprotein that has interaction with *β*2-microglobulin and intracellular TfR1, which is necessary for its stability/trafficking through the endoplasmic reticulum-Golgi pathway and cell surface localization. It is also a binding partner of TfR1 on the cell surface lowering the apparent affinity of the receptor for diferric transferrin (Fe_2_-Tf) and thereby reducing the amount of iron entering cells. The presence of the C282Y mutation has been shown to render the HFE protein unable to interact with *β*2-microglobulin/TfR1 thereby preventing its cell surface expression, whereas the H63D variant interacts normally with TfR1 but reduces the affinity of TfR1 for diferric transferrin to a lesser extent [22–24]. Little is known regarding the role of HFE protein in the regulation of hepcidin. Some authors have proposed that increased transferrin saturation leads to dissociation of HFE protein from TfR1 which subsequently forms a complex with TfR2, which in turn upregulates hepatic hepcidin expression [25]. More recent evidence suggests, however, that HFE protein may not bind to TfR2 [26, 27]. A recent study [28] suggested that the mechanism by which normal HFE protein regulates hepcidin expression is by binding intracellular ALK3 and mediating its cell surface localization. Presumably, C282Y interacts normally with ALK3 but prevents it from reaching the cell surface, whereas interaction of H63D with ALK3 leads to increased ALK3 ubiquitination with the subsequent proteasomal degradation.

One hypothesis consistent with our findings is that differences in baseline levels of hemoglobin reflect differential expression of hepcidin. It is possible, for example, that a proportion of untreated C282Y homozygotes have higher baseline hemoglobin because they produce more hepcidin than those with lower baseline hemoglobin. Higher circulating hepcidin is expected to lead to a greater decrease in the amount of FPN1 on erythroid precursors and thereby to increased iron retention in cells, which in turn will lead to an increase in the availability of iron to enhance hemoglobin synthesis. Subjects with lower circulating hepcidin levels are expected to have higher transferrin saturation, due to increased iron efflux from erythroid precursors, thereby reducing their ability to use transferrin-bound iron as efficiently as those with higher circulating hepcidin levels, potentially explaining the observation that the patient groups with high SF (>1000 *μ*g/L) and with lower mean hemoglobin values had higher mean values of TS (see Table 4). These findings agree with a prior study by Brandáo et al. [29] of 45 C282Y-homozygous patients, in which mean hemoglobin concentrations were significantly lower in patients with high TS (>70%) than in patients with TS between 50 and 70%. In support of this interpretation, iron export from erythroid precursors and enterocytes is shown to be mediated by FPN1B, a product of a splice variant of the transcript for FPN1 that lacks an iron responsive element (IRE) in its 5′ untranslated region and thus is not subject to regulation by intracellular iron, making hepcidin the main negative regulator of FPN1 expression on erythroid precursors [30].

The decreases in posttreatment hemoglobin in subgroups with higher baseline hemoglobin were significantly greater than in subgroups with lower baseline hemoglobin. These differences could not be explained by differences in the number of phlebotomies needed to achieve iron depletion because the subgroups with higher baseline hemoglobin on average needed fewer phlebotomies to achieve iron depletion than the subgroups with lower baseline hemoglobin. Nor could the differences be attributed to other baseline or treatment characteristics such as treatment intensity at the end of therapeutic phlebotomy or the degree of posttreatment SF reduction (Table 4). Several studies in* HFE*-related HH patients have shown that phlebotomy therapy leads to a further decrease in the circulating hepcidin levels or to upregulation of divalent metal transport 1 (DMT1) leading to increased intestinal iron absorption and increased iron release from stores [31, 32]. Thus, it is conceivable that the extent to which phlebotomy-induced iron depletion influences hepcidin expression and/or the amount of iron absorbed may contribute to the observed differences in posttreatment hemoglobin levels in treated patients.

The finding that C282Y-homozygous patients with higher mean baseline hemoglobin had lower phlebotomy requirements to achieve iron depletion than those with lower mean baseline hemoglobin probably reflects the relative amount of iron stored as ferritin and hemosiderin, rather than an effect of baseline hemoglobin per se. The release of iron from hemosiderin has been shown to be slower than that of ferritin [33], probably due to the time required for degradation of hemosiderin. The distribution of iron among parenchymal cells in different tissues may also affect iron mobilization from storage sites.

In the present study, we compared the importance of baseline hemoglobin level as a predictor of phlebotomy requirement for reduction of iron overload with serum markers of iron. Multiple linear regression analysis performed for male patients identified baseline SF as a strong predictor of phlebotomy requirement whereas baseline hemoglobin was found as a poor, albeit independent, predictor. Baseline hemoglobin was, however, not an independent predictor of phlebotomy requirement in the smaller group of female patients.

However, the ability of baseline SF to predict phlebotomy requirements to induce iron depletion in C282Y-homozygous subjects with iron overload is less clear. For example, the amount of iron mobilized as estimated by phlebotomy, assuming that each unit of blood (480 mL) represents 240 mg of iron, in male patients averaged 5.2 g (645 g/124), which is considerably less than 7.5 g (931 g/124) calculated from baseline SF concentrations, assuming that 1 *μ*g of SF is equivalent to 8 mg of storage iron. The corresponding values for female patients were 3.7 g (267 g/72) and 5.2 g (372 g/72), respectively. In comparison, the average amount of storage iron as estimated by phlebotomy in male compound heterozygotes was 2.7 g (49 g/18) compared with 5.1 g (91 g/18) calculated from baseline SF concentrations. These observations, which are in agreement with previous studies [34, 35], suggest that baseline SF levels as an index of iron stores are not able to accurately predict the actual iron status and, thus, the phlebotomy requirement, in* HFE*-related HH patients.

Despite the strong association between the C282Y/C282Y genotype and iron overload, there is so much variability within this group that genotypic information alone is not sufficient to predict the severity of iron overload in any individual subject, suggesting that common variants of other genes involved in the iron metabolism and/or environmental factors modify the penetrance of the C282Y/C282Y genotype in these subjects [36]. Indeed, a polymorphism (p.D519G) in the gene encoding glyceronephosphate O-acyltransferase (GNPAT) was recently identified as one such modifier [37]. The authors studied this polymorphism with regard to its effect on the severity of iron overload and found that 68% (15/22) of C282Y-homozygous males with high-iron phenotypes carried one allele and one male carried two alleles of GNPAT p.D519G. Importantly, the authors provide data indicating that GNPAT p.D519G modulates the severity of iron overload through downregulation of hepcidin expression.

There are a number of limitations of this study. First, our study was retrospective and therefore does not infer causality between variables examined here. Moreover, the retrospective nature of the study limited the interpretation of our findings. Second, data for transferrin saturation were not available for all patients. Third, our study contained few female patients with SF above 1000 *μ*g/L and was therefore not powered to detect true differences. Fourth, we also did not measure hepcidin as no frozen serum samples for assays were available. Finally, although the data presented here can be viewed as hypothesis-generating, they must be followed by confirmation in an independent prospective study. However, to our knowledge, this is the first study in C282Y-homozygous patients evaluating baseline hemoglobin to examine whether this red blood cell parameter is associated with body iron stores (as reflected in SF concentrations) and reporting differences in posttreatment changes in hemoglobin and phlebotomy requirements based on subgrouping patients according to SF and baseline hemoglobin levels.

In conclusion, the results of this study show that treating C282Y-homozygous patients as a homogeneous group or dividing them into just two subgroups according to baseline SF levels may obscure important distinctions which only become evident when patients are further divided into subgroups based on other baseline characteristics. Our results may also suggest that baseline TS levels and hemoglobin levels in C282Y homozygotes may not necessarily run in the same direction.

## Figures and Tables

**Table 1:** Baseline characteristics of the patients (*n* = 214) according to *HFE* genotype.

Genotype groups	Age^*∗*^	SF (*μ*g/L)	TS (%)	Hb (g/L)
Males (*n* = 142)				
C282Y/C282Y (*n* = 124)	47.3 ± 13.0	979 ± 688	78.2 ± 15.5	150.4 ± 10.1
	(20–84)	(316–4000)	(39–100)	(124–172)
				
C282Y/H63D (*n* = 18)	51.0 ± 9.5	632 ± 179^†^	59.2 ± 15.4^†^	153.2 ± 6.7
	(29–69)	(326–967)	(31–88)	(143–169)
Reference ranges		[15–300]	[≤50%]	[130–165]

Females (*n* = 72)				
C282Y/C282Y	54.6 ± 10.3^‡^	646 ± 496^‡^	74.5 ± 16.8	138.2 ± 8.1^‡^
	(31–71)	(210–3100)	(29–100)	(116–156)
Reference ranges		[15–200]	[≤45%]	[115–155]

Results are mean ± SD; values in parentheses are range: minimum–maximum.

^*∗*^Age at the start of treatment.

^†^ *p* < 0.001, comparison between male C282Y/H63D and male C282Y/C282Y.

^‡^ *p* < 0.001, comparison between female C282Y/C282Y and male C282Y/C282Y.

Hb: hemoglobin; SF: serum ferritin; TS: transferrin saturation.

**Table 2:** Association between baseline hemoglobin and other baseline characteristics in C282Y-homozygous males (*n* = 124) and females (*n* = 72).

Variables	Males				Females			
	Age	Log-SF	TS (%)	Log-PH	Age	Log-SF	TS (%)	Log-PH
Base-Hb	−0.29^†^	−0.25^†^	−0.23^†^	−0.37^‡^	0.08	−0.27^†^	0.02	−0.27^†^
Age		0.29^†^	0.01	0.46^‡^		0.07	0.21	0.00
Log-SF			0.45^†^	0.71^‡^			0.34^†^	0.72^‡^
TS (%)				−0.36^‡^				0.29^†^

Base-Hb: baseline hemoglobin; SF: serum ferritin; Log-PH: log number of phlebotomies.

^†^ *p* < 0.05.

^‡^ *p* < 0.01.

**Table 3:** Comparisons of mean ± SD decreases (95% confidence interval), percentage changes in posttreatment hemoglobin from baseline, and the mean ± SD number of phlebotomies among hemoglobin subgroups of C282Y homozygotes and male genotype groups.

	Base-Hb	Post-Hb	MD (95% CI)	% change	Post < Base	Nr-PH
	g/L^†^	g/L^†^	g/L	g/L	*N* (%)	Mean ± SD
Males						
C282Y/C282Y	150.4 ± 10.1	144.4 ± 11.0	−6.0 (−7.7, −4.4)	−4.0	93/124 (75)	21.7 ± 14.8
						
SF <1000 *μ*g/L	151.9 ± 8.4	145.3 ± 10.7	−6.6 (−8.7, −4.5)	−4.2	65/85 (76)	16.0 ± 6.8
SF >1000 *μ*g/L	147.1 ± 12.6	142.2 ± 11.4	−4.8 (−7.7, −1.9)	−3.3	28/39 (72)	34.0 ± 19.4
*p* value	0.014			0.31		<0.001
						
Low SF/high Hb	157.4 ± 4.7	149.0 ± 10.9	−8.4 (−11.4, −5.5)	−5.3	43/52 (83)	14.3 ± 5.1
Low SF/low Hb	143.3 ± 4.9	139.6 ± 7.5	−3.7 (−6.2, −1.2)	−2.6	22/33 (67)	18.8 ± 8.1
*p* value	<0.001			0.038		0.038
						
High SF/high Hb	160.3 ± 5.6	151.2 ± 9.4	−9.1 (−14, −4.3)	−5.7	13/16 (81)	26.7 ± 11.3
High SF/low Hb	137.8 ± 6.2	136.0 ± 8.1	−1.8 (−5.1, −1.5)	−1.3	15/23 (65)	39.1 ± 22.2
*p* value	<0.001			0.014		0.06
						
C282Y/H63D	153.2 ± 6.7	143.4 ± 9.6	−9.9 (−15, −4.9)	−6.5	15/18 (83)	11.2 ± 4.9

Females						
C282Y/C282Y	138.2 ± 8.1	133.1 ± 9.1	−5.1 (−7.3, −3.0)	−3.7	52/72 (72)	15.4 ± 10.9
						
SF <1000 *μ*g/L	139.0 ± 8.1	133.2 ± 9.1	−5.8 (−8.0, −3.6)	−4.2	47/61 (77)	12.3 ± 6.3
SF >1000 *μ*g/L	134.0 ± 6.6	132.5 ± 9.8	−1.5 (−8.5, 5.6)	−1.1	5/11 (45)	32.7 ± 14.7
*p* value	0.059			0.16		<0.001
						
Low SF/high Hb	144.3 ± 5.1	136.3 ± 7.7	−8.0 (−10.5, −5.5)	−5.5	31/35 (89)	12.5 ± 7.5
Low SF/low Hb	131.2 ± 5.3	128.9 ± 9.2	−2.8 (−6.7, −1.0)	−2.1	16/26 (62)	12.0 ± 4.1
*p* value				0.020		0.64
						
High SF/high Hb	142.0 ± 2.0	136.7 ± 3.2	−5.3 (−9.1, −1.5)	−3.7	3/3 (100)	20.7 ± 10.3
High SF/low Hb	131.0 ± 4.9	131.0 ± 11.2	0.0	0.0	2/8 (25)	37.3 ± 13.9
*p* value	0.005			0.49		0.036

^†^Results are mean ± SD.

Base-Hb: baseline hemoglobin; Post-Hb: posttreatment hemoglobin; MD: mean difference between posttreatment hemoglobin and baseline hemoglobin; Post < Base: number (%) of patients with decreased posttreatment hemoglobin compared with baseline values; Nr-PH: number of phlebotomies.

The percentage change is expressed as difference between posttreatment hemoglobin level and baseline hemoglobin level divided by baseline hemoglobin level × 100 and was used as a continuous variable for calculations of the statistical differences between subgroups.

**Table 4:** Baseline and treatment characteristics of hemoglobin subgroups of C282Y homozygotes by gender.

Subgroups	Age	SF (*μ*g/L)	TS (%)	Interval^*∗*^	Post-SF^*∗∗*^
Males					
Low SF/high Hb	43.4 ± 13.3	640 ± 169	74.1 ± 15.2	15.7 ± 6.12	42.1 ± 5.23
Low SF/low Hb	47.2 ± 10.6	685 ± 167	72.5 ± 14.5	16.1 ± 4.10	42.3 ± 4.33
*p* value	0.18	0.25	0.68	0.71	0.83
					
High SF/high Hb	50.5 ± 13.7	1728 ± 931	80.9 ± 12.9	13.6 ± 4.08	42.0 ± 3.76
High SF/low Hb	53.9 ± 12.2	1647 ± 819	91.9 ± 6.6	14.4 ± 3.41	41.7 ± 4.32
*p* value	0.43	0.80	0.002	0.52	0.73

Females					
Low SF/high Hb	54.9 ± 10.2	466 ± 164	75.7 ± 16.4	15.8 ± 5.19	41.9 ± 5.96
Low SF/low Hb	53.5 ± 11.4	480 ± 161	68.6 ± 15.0	17.0 ± 5.03	42.9 ± 4.51
*p* value	0.72	0.62	0.31	0.39	0.48
					
High SF/high Hb	51.7 ± 11.0	1872 ± 375	75.7 ± 24.4	18.7 ± 4.04	43.7 ± 2.31
High SF/low Hb	57.8 ± 7.1	1513 ± 683	91.6 ± 3.8	16.3 ± 4.26	44.1 ± 3.18
*p* value	0.30	0.26	0.18	0.40	0.82

Results are mean ± SD.

^*∗*^Interval: the time interval between the penultimate phlebotomy and the last phlebotomy.

^*∗∗*^Post-SF: posttreatment SF.
